# Folie À Trois: A Case of Shared Delusions Between a Patient, Her Sister, and Another Patient in the In-Patient Psychiatric Unit

**DOI:** 10.7759/cureus.43304

**Published:** 2023-08-10

**Authors:** Allison Zuckerberg, Margaret Carter, Tyler A Barreto, Ruby Barghini

**Affiliations:** 1 Psychiatry, Temple University Hospital, Philadelphia, USA

**Keywords:** schizophrenia and other psychotic disorders, folie a deux, folie à deux, induced delusion, shared psychosis, shared delusion disorder, psychosis

## Abstract

Folie à deux is a rare condition in which a single person (often with a psychiatric disorder) develops a delusion that is shared by another person. Folie à troix is when a delusion is shared by three people. This case report documents the unusual case of an individual who shared delusions with two different people simultaneously. This report inspires questions about this person, her delusions, and what made them so believable to others. It is known that the development of shared delusions most commonly occurs in relative isolation and disproportionately affects individuals with preexisting psychiatric comorbidities. Because of these risk factors, delusions in a psychiatric unit may be even more “contagious” than in the general population. To our knowledge, this case report is the first to document a newly developed delusion shared between two unrelated patients in a single psychiatric unit. While physical separation of patients is the best practice in such cases, a risk-benefit analysis is needed prior to this intervention given the social barriers that may limit such an approach. Further research is needed to diagnose, manage, and optimize treatment for shared delusions in settings such as inpatient psychiatric facilities.

## Introduction

Folie à deux occurs when a “primary” presents with features of psychosis and a “secondary” demonstrates shared psychotic features. Although it is most often seen between two individuals, a primary can influence more than one person in rare cases [1]. This case report illustrates the clinical presentation of one individual sharing a delusion with two different individuals simultaneously, or folie à trois. In the Diagnostic and Statistical Manual of Mental Disorders-IV (DSM-IV), this condition is diagnosed when a delusion develops in an individual in the context of a close relationship with another person(s) who has an already-established delusion, is similar in content to that of the person who already has the established delusion, and the disturbance is not better accounted for by another psychiatric disorder or a mood disorder with psychotic features and is not due to the direct physiological effects of a substance or a general medical condition [2]. In the DSM-V, this was removed and is now described as “delusional symptoms in the partner of an individual with delusional disorder” in the section “Other Specified Schizophrenic Spectrum and Other Psychotic Disorders" [3]. We use this case report to review common themes noted in previous literature and recommend future directions for studies of this rare phenomenon.

## Case presentation

A middle-aged female with a past psychiatric history of schizophrenia, as per the patient’s outpatient medical chart, was brought to the emergency department by police after attempting to trespass into a home that was not hers and harassing the homeowner. She was noted by the police to claim that she owned the entire block of homes, had trillions of dollars, and that there was a single individual she called “the businessman” who was responsible for why she could not provide evidence. She said that it was because he had stolen all of her personal documentation many years ago. At the hospital, the patient was noted to display threatening behavior towards staff, requiring the use of antipsychotics and restraints to complete a full evaluation. The decision was made to involuntarily hospitalize the patient for the management of her psychosis.

When contacting the patient’s sister who she had been living with, it was clear that she also shared similar beliefs regarding the businessman and behaved as if it were reality. Although she had never met the businessman or witnessed anything, she agreed that he had stolen all of her sister’s documents and that he was the cause of her current problems. She denied that her sister was mentally ill, saying that it seemed like her sister was being hospitalized on a misunderstanding and prior hospitalizations were for the same reason. The sister also denied that she herself had a mental illness and repeatedly expressed concern that this person was listening in on the phone calls. Nothing further could be determined about this businessman since the patient said she had no other collateral contacts and throughout the hospitalization, attempts to recontact the patient’s sister were unsuccessful.

The patient was treated with risperidone which improved her affect and behavior, but only minimally improved her delusions. During the hospitalization, she continued to endorse delusions regarding the businessman. One such delusion was that some of the hospital staff were hired by him and went through her personal belongings which were in a locked closet, despite confirming with multiple staff members that this was not the case. Interestingly, after some time the patient befriended another patient hospitalized in the unit. They were observed discussing plans about seeing one another again once they were both discharged. After befriending one another, staff observed the second patient start endorsing a similar delusion about staff going through her possessions, although to a lesser degree. Unfortunately, we were not able to collect information regarding the second patient's admitting psychiatric diagnosis when we had the information to identify her. It cannot be definitively determined whether or not her similar delusions could be attributable to a different psychiatric disorder. However, this delusion emerging after witnessing these two individuals befriend one another suggests a true case of folie a trois occurred while both patients were hospitalized in the unit.

Fortunately, with further development of positive coping skills, the patient reported to staff that if she saw the businessman or someone who worked with him, she would not directly engage and instead continue to pursue legal means to obtain her documentation. The patient was ultimately discharged back to her sister’s home with recommendations to continue risperidone and follow up as an outpatient. Since the patient’s sister stopped answering calls, it could not be confirmed if her delusion continued to be maintained throughout the patient’s hospitalization. Physical separation was considered for the patient and her sister, but unfortunately, the patient had nowhere else to live. Psychological separation from her sister was recommended instead, including reconnecting with outside family members, friends, and social groups like church. Of note, the second patient stopped reporting any delusions regarding staff accessing her personal belongings shortly after the first patient’s discharge.

## Discussion

Since folie à deux (French for “madness of two”), was first characterized in the late 1800s, many have questioned what causes this phenomenon [4]. Although the mechanism is not understood, researchers have attempted to stratify risk factors that contribute to its development. Social isolation has consistently been identified as the most significant risk factor among those affected [5]. Similarly, our patient was noted to be isolated from society. In one encounter, the patient admitted to clinicians that she was too paranoid to use a cell phone. She could not identify any form of social support besides her sister. Schizophrenia has been considered to be the most likely diagnosis in the primary, which also happened to be our patient’s diagnosis [3]. Generally, those with some form of psychiatric illness have been found to be more susceptible to shared psychosis [6]. 

Literature spanning from 1942 to 1993 found that the majority (90.2%) of cases were within dyads of the nuclear family, and evenly distributed amongst married couples, sibling dyads, and parent-child dyads [7], and a more recent review from 2006 estimated as many as 98% of reported cases occurred within the nuclear family [8]. Gender-concordant pairings were also more common [8]. Earlier hypotheses suggest that women are more susceptible than men [3]; however, other studies have not replicated this [7]. Across the PubMed database, there is one report about a patient on a medical floor who shared delusions with another person, but in this, they were related to each other [9]. Shared delusions develop in a wide variety of contexts and may be under-recognized. There is another case report about three people sharing a delusion that developed in the setting of grieving a shared family member [10]. Our case report details a primary and two separate secondaries who had multiple shared demographics outside of a family relationship including age, gender, and race plus the experience of being hospitalized in a psychiatric setting. To our knowledge, no current literature has considered the representation of race or ethnicity in those who share delusions.

Typically, treatment for shared delusions is separation and treatment of all affected individuals. Unfortunately, this is challenging to address when someone affected is not a patient. We did not have access to the primary patient’s sister's medical record including psychiatric history and cannot treat her. While the physical separation between the patient and her sister could be encouraged, the reality is that the patient was homeless otherwise and already had interacted at least once with law enforcement. The risks vs. benefits of separation were weighed and it was agreed that enforcing physical separation was not feasible and would likely worsen the patient’s overall prognosis. Since psychological separation could improve the patient’s delusions, it was recommended that the patient reconnect with people who may not share her delusion. Additional treatment modalities are needed for patients with shared delusions, as separation and treatment of all parties are not always realistic (Table 1).

**Table 1:** Key points and clinical pearls.

Key points	Clinical pearls
Isolation is a key risk factor for shared psychosis	Investigate other risk factors (e.g. race, ethnicity)
Risks vs. benefits must be analyzed for physical separation	Consider psychological separation if physical separation is not feasible or may worsen the prognosis
Psychological separation is a reasonable alternative for shared psychosis	Educate clinicians about psychological separation
Sharing a psychiatric unit may contribute to the development of shared psychosis	Clinicians should be aware of this potential risk factor

## Conclusions

First-degree, gender-concordant family members with a close as well as isolated relationship is a highly prevalent configuration for shared delusions (i.e., the patient and her sister). Further, individuals with psychiatric comorbidities are more likely to share psychosis (i.e., the second patient on the psychiatric unit). How any kind of *folie *happens is unknown, but its propensity to occur in families may be a combination of isolation and other factors yet to be systematically characterized, such as genetics or characteristics of a shared environment. What is unusual about this case is that an unrelated patient in the psychiatric unit also began endorsing delusions similar in content to the patient and her sister. While it is possible the delusion may have been a symptom of a different psychiatric disorder, its temporality suggests folie à trois. In theory, shared experiences within a psychiatric unit, such as confiscation of personal belongings and a high level of supervision, may increase patients’ perception of isolation. It is possible that this phenomenon occurs commonly in psychiatric units where patients have opportunities to formally and informally interact but is underreported or underrecognized. This case inspires questions about what may contribute to affinity and/or relative social isolation of groups. Further research is needed to diagnose, manage, and optimize treatment for shared delusions in contexts such as the inpatient psychiatric setting.
